# Research on Hotspot Discovery in Internet Public Opinions Based on Improved *K*-Means

**DOI:** 10.1155/2013/230946

**Published:** 2013-09-10

**Authors:** Gensheng Wang

**Affiliations:** Electronic Business Department, Jiangxi University of Finance and Economics, Nanchang 330013, China

## Abstract

How to discover hotspot in the Internet public opinions effectively is a hot research field for the researchers related which plays a key role for governments and corporations to find useful information from mass data in the Internet. An improved *K*-means algorithm for hotspot discovery in internet public opinions is presented based on the analysis of existing defects and calculation principle of original *K*-means algorithm. First, some new methods are designed to preprocess website texts, select and express the characteristics of website texts, and define the similarity between two website texts, respectively. Second, clustering principle and the method of initial classification centers selection are analyzed and improved in order to overcome the limitations of original *K*-means algorithm. Finally, the experimental results verify that the improved algorithm can improve the clustering stability and classification accuracy of hotspot discovery in internet public opinions when used in practice.

## 1. Introduction

The rapid development of the Internet exerts a profound impact on the country, society, and individuals and how to effectively master mass data and extract the hotspot information therein have been a problem urgently to be solved in the management of internet public opinions. Solving this problem has an extensive application prospect: first, for individuals, it is an important means to promptly and conveniently obtain the hotspot information in current society; second, for enterprises, it can help enterprises master the most cutting-edge information and hot technology in their fields, increase their competitiveness for enterprises through this method; especially for the country, it can provide important clues for relevant departments of the governments to promptly know about the direction of public opinions in current society, be conductive to the governments to analyze and guide the public opinions, actively guide the healthy development of internet public opinions; meanwhile, help the governments to grasp the problems mostly cared by the people in each period as well as the viewpoints and attitudes on these problems, so as to make scientific and correct decision, keep the society stable, and truly reach the aim that the Internet serves for the society and the people. In the past, public opinions workers rely on manual work to sort the contents on the webpage to discover the hotspot information of the society, not only low efficiency in work, but also easy to be subjectively influenced and make the result deviate from the truth. At present, search engines, to some extent, meet people's demand on rapidly acquiring information needed among massive and messed information; however, its adoption of simple key words matching to find information causes a great deal of redundant and irrelevant contents in search results, results in redundant information overwhelming the information needed, leads to the incomplete analysis on topics of relevant personnel, and makes it difficult to have a comprehensive mastery. The premise for discovering hotspot information by search engines is that analysts know in advance the existence of such topics, so such method is obviously lagging and it is not good for discovering new problems, easy to miss the best timing to solve problems, making the problems spread and difficult to be controlled. Therefore, if the real-time hotspot information in a period is to be obtained and the internet hotspot topics in current society are to be periodically discovered, automatic solutions are becoming a valuable research orientation.

## 2. Literature Review

At present, the study on hotspot discovery of internet public opinions at home and abroad mainly focuses on such two aspects as internet information processing and data mining. (1) In the aspect of internet information processing, the main research contents of scholars at home and abroad include word segmentation technology, measuring of multidimensional vector space on article theme [1]. (2) In the aspect of internet data mining, contents involved are information acquisition of public opinions, automatic classification, automatic clustering, and so forth, and this kind of methods has obtained certain achievements. For instance, Hamerly and Elkan, on the basis of analyzing the shortages of original *K*-means and its reasons, put forward a new model to mine and analyze internet public opinions information, and illustrated the application of text mining in the analysis of internet public opinions [2]; Kristina analyzed the basic situation of internet public opinions and designed an analyzing model of internet public opinions based on themes [3]; Andreas combined the advantages of comprehensive partitional clustering and agglomerate clustering and put forward an incremental hierarchical clustering algorithm and applied it to hot topic discovery in internet public opinion [4]; Wagstaff and Rogers combined natural language processing with information retrieval technology and put forward a very effective single-granularity topic identification method as to the event features [5]; Ya designed a hotspot events discovery system which is geared to the needs to internet news coverage and able to automatically find the hotspot events on the internet within any period [6]; Bradley and Managasarian, according to the demands on the analysis of internet public opinions, built the discovery and analysis system of internet public opinion hotspots problems based on clustering [7]. As for mass internet public opinion information, how to improve the effectiveness and efficiency of analysis and processing as well as the accuracy and efficiency of the analysis of internet public opinion hotspots remains a hotspot for current research.

Currently, domestic and overseas studies on the clustering methods of internet public opinions are mainly divided into the following categories: partitional clustering, hierarchical clustering, clustering based on density, artificial neural network clustering, clustering based on internet, and so forth in which clustering is widely applied. According to different objects, application fields, and aims of clustering, there are specific requirements on the quality, efficiency, and result visualization degree of clustering for clustering methods. Hence, proper clustering algorithm shall be selected as required by specific conditions, among which as to text clustering, *K*-means clustering, due to its features like increment, batch processing, speediness, and efficiency, as well as its advantage in applicable to dynamically process mass data of internet media information, is widely applied in the detection of internet hotspot topics. However, the clustering quality in *K*-means algorithm relies too much on the initial number of clusters and initial clustering centers, which shall be conquered in actual application.

*K*-means algorithmis one of the best information clustering methods in data mining which can extract and find new knowledge. But it is found that *K*-means algorithm used in processing the data of isolated points has great limitations [6–8]. The paper tries to present some improvements to overcome these limitations and takes advantage of powerful classification ability of the algorithm to discovery hotspot in internet public opinions.

## 3. Text Preprocessing

Hotspot discovery depends on website text clustering which can be described as a given text set *D* = {*d*_1_, *d*_2_,…, *d*_*n*_}, eventually get a cluster's set *C* = {*C*_1_, *C*_2_,…, *C*_*n*_}, ∪_*i*=1_^*K*^ *C*_*i*_ = *D* derive for all *d*_*i*_ (*d*_*i*_ ∈ *D*), ∃*C*_*j*_ (*C*_*j*_ ∈ *C*) and *d*_*i*_ ∈ *C*_*j*_, and also make the objective function *Q*(*C*) reach the minimum or maximum value, of which *n* is total text number, *K* is final clustering number, and *C*_*j*_∩*C*_*i*_ ≠ *ϕ*, *j* ≠ *i*.

### 3.1. Characteristic Selection and Expression of Website Text

Vector space model (VSM) is commonly adopted to express each text. In this model, each text *d* is considered as a vector in a vector space. *tfidf* is used as a measure of characteristic vector in this paper, and this measure gives the weight of each word *t*. See (1) for the calculation of the weight:

(1) $$\begin{array}{c} tfidf\left(d,t\right)=tf\left(dt\right)*\text{lo}{\text{g}}_{2}\frac{N}{df\left(t\right)}. \end{array}$$

In (1), *tf*(*d*, *t*) is the word frequency of word *t* in the text *d*, *df*(*t*) is all the text numbers of word *t* contained in the text set *D*, and *N* is total text number. After the characteristic selection, text *d* ∈ *D* is the form of the vector, and the value of each dimension is the corresponding *tfidf*(*d*, *t*) weight value, so the text can be expressed as follows:

(2) $$\begin{array}{c} d=\left\{\left(t_{i},tfidf\left(d,t_{i}\right)\right)\;|\;1\leq i\leq m\right\}, \end{array}$$

of which *t*_*i*_ is the lexical entry and *m* is the dimension of the characteristic vector. However, after the characteristic selection, *m* is still very large, thousands of dimensions at least and tens of thousands of dimensions at most while nonzero word frequency of each corresponding text vector is very few, which makes text VSM show the high dimension.

### 3.2. Definition of Similarity

In this paper, cosine distance is used to measure the similarity between the website texts and defines the similarity of two texts *d*_1_ and *d*_2_ as follows:

(3) Sim(d1,d2)=cos⁡⁡(d1,d2)=(d1∗d  2  )(norm⁡(d1)∗norm⁡(d2)).

In order to reduce the impact of different length of the texts on calculating the text similarity, each text vector has been integrated to the unit length. See (2):

(4) $$\begin{array}{c} d=\frac{d}{||d||}=\frac{\left\{tfidf\left(d,t_{1}\right),fidf\left(d,t_{2}\right),\ldots ,fidf\left(d,t_{m}\right)\right\}}{\sqrt{\left\{tfidf{\left(d,t_{1}\right)}^{2},fidf{\left(d,t_{2}\right)}^{2},\ldots ,fidf{\left(d,t_{m}\right)}^{2}\right\}}}. \end{array}$$

Thus, ||*d*|| = 1 and the similarity of the cosine is the dot product of two text vectors; that is, Sim (*d*_1_, *d*_2_) = *d*_1_ · *d*_2_.

## 4. Derivation of Hotspot Discovery Algorithm

### 4.1. *K*-Means Algorithm Principle

**Figure 1:**
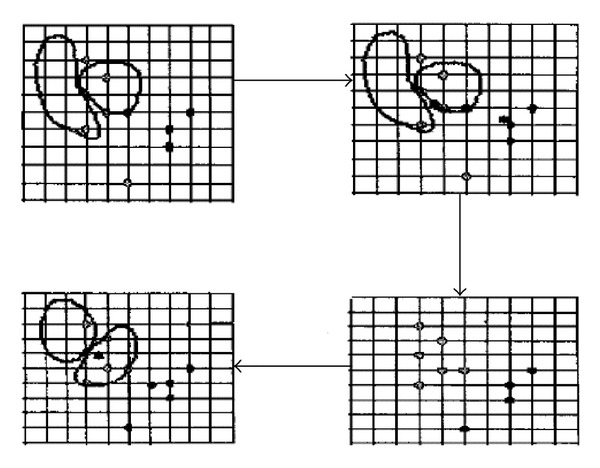
The procedures of *K*-means algorithm.

Steps for *K*-means clustering algorithm are as follows [8] (see Figure 1):

1. Select *n* objects as the initial cluster seeds on principle;
2. Reassign each object to the most similar cluster in terms of the value of the cluster seeds;
3. Update the cluster seeds; that is, recompute the mean value of the object in each cluster, and take the mean value points of the objects as new cluster seeds.
4. Repeat (2) and (3) until no change in each cluster.

### 4.2. Limitation of *K*-Means Algorithm

When *K*-means algorithm is used to cluster data, the stability of the clustering results is still not good enough; sometimes, the clustering effect is very good (when the data distribution is convex-shaped or spherical), while sometimes the clustering results have obvious deviation and errors, which lies in the data analysis. It is unavoidable for the clustered data to have isolated points, referring to the situation that a few data deviate from the high-dense data in intensive zone. The clustering mean point (geometrical central point of all data in the category) is used as a new clustering seed for the *K*-means clustering calculation to carry out the next turn of clustering calculation, while under such a situation, the new clustering seed might deviate from the true data intensive zone and further cause the deviation of the clustering results [9]. Therefore, it is found that using *K*-means algorithm to process the data of isolated points has a great limitation.

### 4.3. Improving of *K*-Means Algorithm Principle

The original *K*-means algorithm selects *K* points as initial cluster centers, and then the iterative operation begins. Different selection of initial point can achieve different clustering result. For the reduction of the clustering result's dependence on the initial value and the improvement of the clustering stability, better initial cluster centers can be achieved by the search algorithm of the cluster center [9, 10].

**Figure 2:**
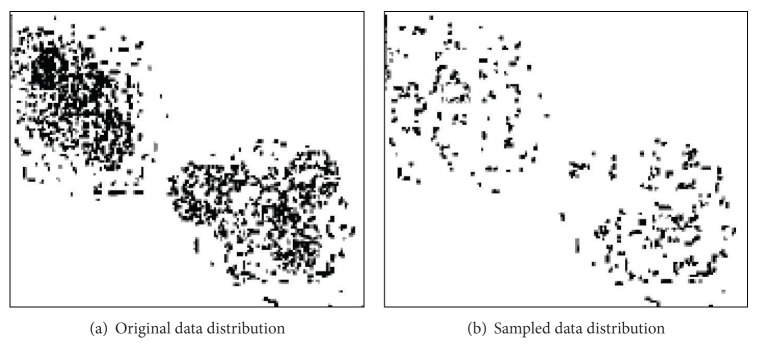
Comparison of data distribution before and after sampling.

In the search process, the sampled data tries to be undistorted and is able to reflect the original data distribution through the random data sampling, as shown in Figure 2, among which, (a) original data distribution, (b) sampled data distribution.

The sampled data and the original data are clustered by *K*-means algorithm, respectively, and little change of final cluster centers is found. Therefore, the sampling method is suitable for the selection of the initial cluster centers. In order to minimize the sampling effects on the selection of the initial cluster centers, the sample set extracted each time should be able to be loaded into the memory and do best to make the sum of the sample sets extracted *J* times equivalent to the original data set. Each extracted sample data is clustered by *K*-means algorithm and one group of cluster center is produced, respectively; the samplings *J* times produce *J* groups of the cluster centers in all, and then the comparison of clustering criterion function values is conducted for *J* groups of cluster centers, and one group of minimum cluster center in *J*_*c*_ value is given as the optimal initial cluster center.

For the protection against segmenting large clusters into small clusters by the criterion function, the algorithm takes the initial cluster as *K*′and *K*′≻*K*. According to the quality requirements and the time, *K*′ value does the compromise selection. Larger *K*′ value is able to expand the solution search scope, and the phenomenon of no initial value near certain extremal vertexes is diminished. The utilization of the searched initial cluster center clusters the original data by another *K*-means algorithm and outputs *K*′ cluster centers, and then the reduction of each cluster quantity to the specified *K* value is studied.

### 4.4. Improving the Selection of Initial Classification Centers

The basic idea of new selection method of initial cluster centers is based on the assumption that the distribution of the website text sets has been known; a good initial cluster center should satisfy the following rules in the paper.

1. The selected initial centers belong to different clusters, respectively; that is, any two initial centers cannot be the same cluster;
2. The selected initial cluster centers should represent this cluster, that is, be as close as possible to the cluster centers. To select *K* texts as initial cluster centers and at the same time ensure that *K* texts just belong to different clusters, such strict constraints are difficult to be achieved through random sampling as much as possible, so it is thought that in order to minimize the sampling's effect on initial cluster centers, *m* times of samplings are taken and the sample size is *n*/*m*, of which, *n* is the number of the text in the text sets, the value of *m* is that each sample size should be put into the main storage and as far as possible satisfies the fact that the sum of the samples taken for *m* times is equivalent to the original text set. Each sample text taken is clustered by *K*-means algorithm to produce a group of text clusters with *K* cluster centers, respectively; *m* times of sampling operation produce *m* × *K* cluster centers in all, and then agglomerative hierarchical clustering algorithm single-link algorithm is used to do the clustering to obtain *K* clusters, of which, the average value is the final *K* initial cluster centers. Different from the division strategies taken by *K*-means algorithm, the agglomerative hierarchical clustering algorithm does not exist in the selection of the initial cluster centers. It regards each text as a cluster at first; the text is the centre of this cluster, and each step of clustering combines the two most similar clusters into a cluster until all the texts are integrated into a cluster or only *K* clusters. With clustering, the similar text is integrated into a cluster gradually and the hierarchical clustering is able to automatically generate different hierarchical clustering model.

In the combination of agglomerative hierarchical clustering algorithm and *K*-means algorithm, a hierarchical clustering algorithm based on *K*-means is addressed to select the initial cluster centers; that is, the cluster centers produced by *K*-means method restrain the agglomerative space of the agglomerative hierarchical clustering algorithm. The selection method of the initial cluster centers is generally described as follows.

1. *m* times of sampling are taken for the text sets, which are divided into *m* sample sets {*S*_1_, *S*_2_,…, *S*_*m*_}.
2. Each sample set performs *K*-means algorithm, respectively, to produce *m* groups of *K* cluster centers.
3. Another clustering is done for *m* × *K* cluster centers by the agglomerative hierarchical clustering algorithm (single-link algorithm is used here) until only having *K* clusters, and the average value of each cluster is taken as the initial cluster centers of next step of *K*-means algorithm.

From the previous algorithm, it is seen that the text set of the sample taken is smaller than the original text set, so the search process amount of the initial cluster centre is less, the iterative number is less, and the speed is faster; at the same time, it is also ensured that the final cluster centers belong to different clusters and have adequate representation.

The specific algorithm flow used in the paper can refer to the reference [8].

## 5. Experimental Verification

### 5.1. Data Acquisition and Preprocessing

 Verification data acquisition and preprocessing in this paper mainly include the following steps. (1) Public opinions data acquisition adopts web search technology, traversing the entire Web space within designated scope to collect all kinds of public opinions information, establishing indexes of acquired information through indexer, and save in the index database. Objects of data acquisition are mainly each major web portals, BBS, blogs, and so forth. (2) Word segmentation processing of website text: public opinions information acquired are unstructured data, which shall be preprocessed. Word segmentation study of Chinese language has been mature. This thesis adopts the Chinese Lexical Analysis System of Institute of Computing Technology (ICTCLAS). (3) Text features abstraction: the aim of selecting features is to further filter works with no much amount of information and less influence on the discovery of public opinions hotspots, reaching the effect of dimension reduction of website feature vector, so as to improve the processing efficiency and reduce the complexity of calculation. Form of dimension reduction adopted in this thesis to build evaluation function of webpage theme through statistical methods, evaluating each feature vector and choosing words meeting the preset threshold as the feature item of webpage; (4) Feature representation: this paper adopts vector space model (VSM) to indicate public opinions information; here omit the specific forms.

### 5.2. Experimental Results

Considering that news is paid high attention in Internet information and it is easy to collect information, this paper takes internet news as verification data. First, randomly choose 8919 pieces of news among the politics news on December 1, 2012 to December 15, 2012 as the test samples obtained by features words of webpage cluster. As webpage comes from real website, webpage data have certain complexity and randomness. After word segmentation processing, there are 68213 words in total; 52173 words are obtained after stop words processing to carry out information for subsequent calculation; take top 10% words, that is, 6512 words, as the feature vector of webpage text. Test results are as shown in Tables 1, 2, and 3.  Table 1 is the statistical table of vocabulary and word frequency with large information gain value; Table 2 is the statistical result of news hotspots themes; Table 3 is the clustering performance comparison of the algorithm in this paper and ordinary *K*-means [8].

In Table 3, *F*_1_ means *F*-measure value, and *F*_1_ distribution is wildly used to illustrate the performance of different algorithms [3–6, 11]. Using the data introduced previously and specific calculation items can be seen in [8]. It can be seen from Table 3 that there is poor stability in the clustering results obtained by ordinary *K*-means algorithm and scattered *F*-measure value, but the improved clustering algorithm has better stability of the clustering results, more concentrated *F*-measure value, and higher *F*-measure average value. The experiment shows that the improved clustering algorithm improves its accuracy and stability greatly. In the use of ordinary *K*-means algorithm, *F*-value of the clustering results scatters from 0.60 to 0.75; in the use of the improved algorithm, the stability of its value is from 0.75 to 0.85.

## 6. Conclusion

Nowadays, internet is becoming the main channel for people to obtain and release information, the guiding role of internet public opinions information is larger and larger; it has aroused wide attention in the industry how to carry out public opinions gathering and hotspots discovery on the basis of information acquisition of Internet public opinions as well as track and analyze the hotspots to guarantee the information security. Under such background, this paper, based on analyzing the advantages and disadvantages of all kinds of clustering algorithms, chooses *K*-means clustering as the website text clustering model and puts forward a new discovery algorithm of internet public opinions hotspots through improving its shortcoming of sensitivity to initial number of clusters and initial clustering centers. The test illustrates the applicability and reliability of method in this paper. The next study shall be focused on clustering of features of internet information text, for the sake of final realization of clustering algorithm applicable to all the languages.

## Figures and Tables

**Table 1:** Parts statistics of feature vector word frequency.

Diaoyu Islands	American	China	Syria	Russia	Military	Japan	Shinzo Abe	Obama	Hugo Chavez
12156	8973	9987	4612	3416	1256	3421	1281	2521	1452

**Table 2:** Parts statistics of news hotspots themes.

News themes	The number of pages	Feature words
Diaoyu Islands	1524	Sovereignty, Shinzo Abe, island Purchase, Escort, Military, Fighter, American, China, Japan
Syria Crisis	642	The opposition, Muslim, Shiite, Sunnite, BaShaEr, Antiterrorism, Iran, Russia, American, the Arab league

**Table 3:** Algorithm performance *F*_1_ comparison of different algorithms.

*F*_1_	*F*_1_ typical value	*F*_1_ of original *K*-means algorithm falling into the experimental frequency of this interval	*F*_1_ of improved *K*-means algorithm falling into the experimental frequency of this interval
[0.15,0.25]	0.20	1	0
[0.25,0.35]	0.30	2	0
[0.35,0.45]	0.40	2	0
[0.45,0.55]	0.50	4	0
[0.55,0.65]	0.60	5	0
[0.65,0.75]	0.70	7	9
[0.75,0.85]	0.80	2	11
[0.85,0.95]	0.90	1	8
[0.95,1.00]	1.0	0	0
